# Association between outdoor physical activity and positive mental health in adolescence: estimating the mediation effect of autonomy, competence and relatedness

**DOI:** 10.1186/s12966-025-01847-z

**Published:** 2025-11-24

**Authors:** Chloé Drapeau, Lars Lenze, Corentin Montiel, François Gallant, Mathieu Bélanger, Isabelle Doré

**Affiliations:** 1 École de kinésiologie et des sciences de l’activité physique, Faculté de médecine, Université de Montréal, Montréal, QC Canada; 2 Centre de recherche du Centre hospitalier del′Université de Montréal, Montréal, QC Canada; 3 Institute of Social and Preventive Medicine, University of Bern, Bern, Switzerland; 4 Département de médecine sociale et préventive, École de santé publique, Université de Montréal, Montréal, QC Canada; 5 Department of Family Medicine, Dalhousie University, Halifax, NS Canada; 6 IMPACTS Laboratory, Centre de Formation Médicale du Nouveau-Brunswick, Moncton, NB Canada; 7 Département de médecine de famille et de médecine d′urgence, Université de Sherbrooke, Sherbrooke, QC Canada; 8 Réseau de Santé Vitalité, Moncton, NB Canada

**Keywords:** Outdoor, Physical activity, Positive mental health, Flourishing, Basic psychological needs, Adolescents, Mediation analyses

## Abstract

**Background:**

Both nature and physical activity practice have been identified as positive contributors to mental health and well-being. Engaging in outdoor physical activity (OPA) likely combines these benefits. However, the mechanisms through which these associations operate remain unknown. Since OPA can promote the satisfaction of the basic psychological needs of autonomy, competence and relatedness and basic psychological needs are associated with positive mental health, they could represent a mediator in the OPA-positive mental health association. The aims of this study are to 1) estimate the association between OPA and positive mental health in adolescents and 2) examine whether satisfaction of autonomy, competence and relatedness mediate this relationship.

**Methods:**

Data from the MATCH longitudinal study were used to examine these objectives in young (14–15 years), middle-age (15–16 years) and older (16–17 years) adolescents. OPA and satisfaction of the basic psychological needs of autonomy, competence and relatedness were self-reported three times per year. Positive mental health was self-reported once per year. Linear and logistic regression models (objective 1) and mediation analyses based on counterfactual definitions of natural direct (NDE) and natural indirect (NIE) effects (objective 2) were performed, adjusting for age, gender, puberty stage, and neighborhood income.

**Results:**

No association was found between OPA frequency and positive mental health in young adolescents (OR [95% CI] = 1.10 [0.69, 1.75]); however, a positive association is observed in middle-age (OR [95% CI] = 1.99 [1.11, 3.57]) and older (OR [95% CI] = 3.40 [1.25, 10.09]) adolescents. Mediation analyses suggest that only relatedness may mediate the OPA-positive mental health association among middle-age adolescents.

**Conclusions:**

Results indicate that OPA may relate differently to positive mental health across adolescence and that underpinning mechanisms need to be further investigated.

**Supplementary Information:**

The online version contains supplementary material available at 10.1186/s12966-025-01847-z.

## Introduction

Adolescence is a transition period marked by numerous and significant changes that increase the vulnerability of youth to mental health challenges. With as many as one in seven adolescents living with mental health conditions, youth mental health is recognized as a public health priority [51, 75]. Moreover, 70% of individuals living with a mental illness experience their first symptoms before the age of 18 [47]. Key changes during adolescence include puberty, brain development, cognitive skills, changes in the social environment [4], exposure to adversity, pressure to conform with peers, identity exploration and influence of media and gender norms [75].

Mental health comprises two distinct but interrelated dimensions: positive mental health and mental illness [29, 32, 33]. Positive mental health is defined as “a state of well-being that allows us to feel, think, and act in ways that enhance our ability to enjoy life and deal with the challenges we face” [53]. High levels of positive mental health serve as a vital resource, supporting adolescents to adapt, manage their emotions, solve problems and build meaningful relationships [75], and act as a protective factor for mental disorders [34]. According to Keyes’ model [32, 33], mental health is categorized as *flourishing*, *moderate* and *languishing*. Flourishing refers to a high level of positive mental health, combining feeling good (i.e., hedonic well-being) and functioning well (i.e., eudaimonic well-being) whereas languishing reflects low levels of both emotions and functioning, and moderate lies between flourishing and languishing [32, 33]. A Canadian study reported that the proportion of adolescents with flourishing mental health dropped from 47% in 2016–2017 to 37% in 2022–2023 and that nearly twice as many adolescent girls than boys report languishing mental health (15.3% vs. 8.3%) [66]. These findings underscore the urgent need for strategies to foster positive mental health in adolescents.

Physical activity is widely recognized as a key strategy in promoting adolescents positive mental health and preventing or alleviating symptoms of mental illness [3, 6, 10, 41, 50, 54, 71]. Specifically, physical activity among adolescents is associated with reduced anxiety [50] and depressive symptoms [6, 10, 46, 50], reduced occurrence of depression disorder [3, 6, 10], and reduced stress, negative affect and psychological distress [54]. Physical activity during adolescence is also associated with positive mental health [12–14], psychological well-being [17, 41, 54], enhanced self-image, life satisfaction and greater happiness [54].

Exposure to nature has also been associated with numerous mental health benefits, including reduced stress symptoms in both adults and adolescents [11, 19, 23, 60, 65], improved mood [19, 60], improved subjective well-being including increased happiness, improved life satisfaction and greater vitality [48] in adults, heightened resilience [65], and enhanced health-related quality of life [65] in adolescents. Being in nature has also been shown to reduce symptoms associated with attention deficit disorder/hyperactivity disorder in adolescents [65], lower levels of depression [19, 40] and anxiety [2, 19, 40, 60], decreased levels of anger and aggressiveness [60], increased self-esteem [60] and improved sleep quality [19] in adults. Most evidence is based on adult samples, with fewer studies focusing specifically on adolescents.

Mounting evidence highlights that mental health benefits of being physically active may be more important for physical activities performed outdoors (e.g., bicycling, skiing, rock climbing) compared to physical activity indoors [16, 31, 49, 58, 73]. Engaging in outdoor physical activity (OPA) likely combines the benefits of physical activity with exposure to nature, potentially amplifying mental health benefits. A UK study indicated that adults engaging in OPA experienced significantly lower anxiety levels and greater feelings of connection to nature compared to those engaging in indoor physical activities [37]. Outdoor environments are considered more restorative than indoor settings [28], aligning with various theories on the health benefits of nature. Han [27] found that a 15-min session of low- to moderate-intensity physical activity in an environment with at least 40% visible greenery had higher positive effects on emotions and attention compared to exposure to nature alone. Similarly, Thompson Coon and colleagues [64] reported that physical activity in natural environments, compared to indoor physical activity, was associated with improved psychological outcomes, including enhanced positive emotions and reduced negative emotions, greater feelings of energy, revitalization and enjoyment. Lahart and colleagues [36] found that physical activity in nature had positive effects on affect and perceived effort compared to indoor physical activity in adults. Additionally, a recent meta-analysis indicates that outdoor activities have the largest positive impact on cognition when compared to activities practiced in any other context [43].

Several mechanisms (e.g., neurobiological, psychosocial, behavioural mechanisms) have been proposed in theoretical models to explain the mental health benefits of physical activity and exposure to nature on mental health [18, 41]. Psychosocial mechanisms linking physical activity and mental health can be examined through the lens of the Basic Psychological Needs Theory, a sub-theory of Self-Determination Theory. This framework posits that satisfaction of the basic psychological needs of autonomy (perceptions of choice, volition and endorsement of one’s behaviour), competence (perceptions of mastery and personal ability) and relatedness (connection with others) [55] can promote internal motivation, pleasure and self-regulation of behaviours, as well as optimal well-being in individuals [55]. Furthermore, Ryan and Deci [55] argue that the psychological needs are interdependent. Satisfaction of these basic psychological needs has been suggested as a potential mediator of the association between physical activity and subjective well-being [39], mental health [14, 26, 63, 78], positive affect and greater participation in school physical education classes [72]. Previous studies also suggest that being in nature contributes to satisfying the psychological needs of autonomy, competence and relatedness, and as such have positive effects on mental health [9] and psychological well-being (positive affect, life satisfaction, meaning in life) [77].

Similarly, satisfaction of these needs could potentially explain the benefits of OPA on mental health which has been overlooked in empirical studies. In fact, we hypothesize that there is a combined effect of the physical activity-mental health mechanisms and the nature-mental health mechanisms. Both physical activity and being in nature could promote the satisfaction of the basic psychological needs. OPA could potentially reinforce the psychological needs of autonomy, competence and relatedness by allowing individuals to choose their own behaviours and activities (autonomy), experience a sense of accomplishment and achievement (competence) and foster meaningful connections with others (relatedness). Therefore, it is possible that the basic psychological needs mediate the hypothesized relationship between OPA and mental health.

Despite the evidence supporting the benefits of exposure to nature and physical activity on mental health, few studies have specifically examined OPA in youth populations [61], leaving a gap in understanding whether OPA could potentially foster adolescent positive mental health as well as underpinning mechanisms. Given the tumultuous nature of adolescence, developing a better understanding of how behavioural strategies (e.g., OPA) are associated with mental health across specific phases of adolescence (early, middle, late) is of considerable theoretical and practical importance and may inform the design of tailored interventions aimed at improving well-being and promoting higher levels of positive mental health in this population. Our study focuses on positive mental health rather than mental illness. Specifically, we examine whether and how OPA is associated with positive mental health. Therefore, this study aims to 1) estimate the association between exposure to OPA and positive mental health in adolescents and 2) examine whether satisfaction of autonomy, competence and relatedness are mediating variables that explain this association.

## Methods

### Participants and procedures

We used data from the Monitoring Activities of Teenagers to Comprehend their Habits (MATCH) study, an ongoing longitudinal study investigating physical activity participation from childhood to emerging adulthood [1]. A total of 929 participants from 17 French and English schools from different socioeconomic neighborhoods located in rural and urban areas in New Brunswick (Canada), were recruited. Participants completed self-report questionnaires every four months from grade 5 or 6 until the end of grade 12 (2011–2019, cycles 1–24). The questionnaire was then repeated in 2020–2021 (cycle 25) and every following year (2021–2022, cycle 26; 2022–2023, cycle 27; 2023–2024, cycle 28). No data for positive mental health was available before cycle 16. Therefore, data for the current analysis were drawn from cycles 13 to 22, which represent periods when participants were young (age 14–15, cycles 13–16), middle-age (age 15–16, cycles 16–19) and older (age 16–17, cycles 19–22) adolescents. To be included in analysis, participants needed to provide complete data for all study variables for the specific age-group.

The MATCH study was approved by the ethics review board of the Université de Sherbrooke and all participants provided written informed assent and their parents provided written informed consent. Detailed methods of the MATCH study are reported elsewhere [1].

### Study variables

#### Outdoor physical activity (OPA)

At each data collection cycle, participants reported physical activities practiced outside gym classes within the past four months using a 36-item physical activity checklist. The 36-item physical activity checklist includes all activities commonly practiced by youth in the MATCH study region [7] and activities included in similar validated lists [8, 30, 35, 56]. From the list of 36 activities, the research team contributed to OPA categorization. Multiple steps were taken, such as independent classification by each author, meetings to discuss and email exchanges to summarize decisions and to obtain consensus. To be consistent with the literature, the authors identified OPA as a physical activity, practiced in an open environment, in a dynamic and harmonious relationship with elements of nature [23]. Through this process, the following 13 physical activities were identified as OPA: *skateboarding or scooter, bicycling, golfing, downhill skiing or snowboarding, cross-country skiing, other snow sports, skipping rope, outdoor chores, in-line skating, outdoor play, rock climbing, kayak/canoe* and *other water sports*. Participants self-reported the frequency of their participation in each OPA which was coded numerically to represent weekly frequency: *never* = 0, *once per month or less* = 0.125, *2–3 times per month* = 0.625, *once per week* = 1, *2–3 times per week* = 2.5, *4–5 times per week* = 4.5, *almost every day* = 6.5. The OPA variable was computed by summing the frequencies for the 13 OPA frequencies at each cycle, and a dichotomous OPA variable (*low* or *high*) was created for analyses. Participants were retained if they participated in at least two of the three data collection cycles over the year (cycles 13–14–15, cycles 16–17–18, cycles 19–20–21), which controls for seasonality of OPA participation at each time point (T1-T2-T3).

#### Autonomy

Perceived autonomy was assessed with the 7-item autonomy subscale of the Basic Psychological Needs in Life Scale (BPNLS) [20]. The original scale was adapted to the physical activity context by including the term “physical activity” in the following question: *“The following statements represent different feelings people have when they engage in physical activity. Using the scale provided, please answer the following questions by considering how YOU TYPICALLY feel when participating in physical activity”*. The items were also adjusted according to the physical activity context (e.g., *“When I participate in physical activity, people I interact with regularly tend to take my feelings into consideration”*). Participants responded to each item using a 7-point Likert scale ranging from 1 (*not at all true*) to 7 (*very true*). The score was calculated based on the mean of the four positively worded items and was used as a continuous variable in the analyses. The three negatively worded items were removed due to a better model fit according to a study using the MATCH database [24]. Based on a previous study using MATCH data, the subscale has demonstrated good internal consistency (Cronbach’s α = 0.89) [14]. Cycles 15, 18 and 21 were used for the analyses (T3).

#### Competence

Perceived competence was assessed with the 6-item subscale of the Intrinsic Motivation Inventory (IMI) [44]. The same adaptation as for the autonomy subscale was conducted and items were also adjusted according to the physical activity context (e.g., *“I think I am pretty good at physical activity”*). Participants responded to each item using a 7-point Likert scale ranging from 1 (*not at all true*) to 7 (*very true*). The score was calculated based on the mean of the five positively worded items and was used as a continuous variable in the analyses. Similar to the autonomy subscale, the negatively worded item was removed [25]. Based on a previous study using MATCH data, the subscale has demonstrated good internal consistency (Cronbach’s α = 0.92) [14]. Cycles 15, 18 and 21 were used for the analyses (T3).

#### Relatedness

Perceived relatedness was assessed with the Relatedness to Others in Physical Activity Scale (ROPAS) [74], which consists of six items scored on a scale ranging from 1 (*false*) to 6 (*true*). The score was calculated based on the mean of the six items and was used as a continuous variable in the analyses. Although the ROPAS was developed for an adult population, the validity and reliability of the scores have been demonstrated for adolescents [57]. Based on a previous study using MATCH data, the subscale has demonstrated good internal consistency (Cronbach’s α = 0.96) [14]. Cycles 15, 18 and 21 were used for the analyses (T3).

#### Moderate-to-vigorous physical activity (MVPA)

In sensitivity analyses, we included MVPA as an additional potential mediator. MVPA was calculated as the mean of two items assessing the number of days per week participants engaged in at least 60 min of physical activity: one referring to the past seven days and the other to a typical or usual week. This composite measure is commonly used in studies based on the MATCH dataset. It has acceptable test–retest reliability (ICC = 0.77) and is associated with accelerometer-measured MVPA (*r* = 0.40) (e.g., [14, 21]).

#### Positive mental health

Mental health was assessed using the Mental Health Continuum–Short Form (MHC-SF) [32], which includes 14 items and three subscales measuring mental health’s emotional (3 items), social (5 items) and psychological (6 items) well-being. Participants rate how often they felt each state on a 6-point Likert scale ranging from 0 (*never*) to 5 (*all the time*). A dichotomous mental health variable (*flourishing* or *not flourishing*) was created. Mental health was categorized as *flourishing* if the participants responded 5 (*all the time*) or 4 (*most of the time*) to at least one of the three emotional well-being items and at least six of the 11 positive functioning items (social and psychological well-being) [12, 33]. In this study, if three or fewer items were missing, they were replaced with the means of the other valid scale items before categorizing this variable. If more than three items were missing, the overall score was not computed, and the variable was declared missing. Based on previous studies using MATCH data, the MHC-SF demonstrated high internal consistency for the three subscale (Cronbach’s α = 0.90 to 0.94) and the total scale scores (Cronbach’s α = 0.97) [14, 15]. Cycles 16, 19 and 22 were used for the analyses (T4).

#### Covariates

Causal mediation analyses require the inclusion of confounders of exposure-outcome, exposure-mediator and mediator-outcome associations [67, 70]. Potential confounders of the associations between exposure to OPA (exposure), needs of autonomy, competence and relatedness (mediators) and flourishing mental health (outcome) have been identified in the literature and include age, gender, puberty stage, and neighborhood income. Given that participants age varied within the same adolescent group (young, middle-age or older), age was included as a potential confounder. Cycles 13, 16 and 19 were used for the analyses (T1).

Participants self-reported their *gender* (*boy, girl, other*). *Age* (years) was derived from the difference in time between the date of data collection and participants’ reported date of birth. *Puberty stage* was assessed using the Pubertal Development Scale (PDS) and was classified into Pubertal Categories (PC) using the items for boys and girls outlined by Carskadon and Acebo [5]. The PDS represents the mean score of the items according to gender (*boy* or *girl*), while the five PC (*prepubertal*, *early puberty*, *midpubertal*, *late puberty* and *postpubertal*) are determined using the Description of the Pubertal Category variable table [5]. *Neighborhood income* was determined by matching participants’ self-report 6-digit postal codes with the mean income of individuals aged 15 and over in 2011 in each participant’s neighborhood, as proposed by the National Household Survey (NHS) [62]. Participants were then grouped into neighborhood income groupings using terciles cut-offs (*low*, *moderate* and *high*).

### Data analysis

Descriptive statistics were conducted to assess the distributions, identify outliers and compute frequencies, proportions, means, standard deviations, medians and interquartile ranges. These descriptive statistics were also used to compare participants included and those excluded from the analyses. A multivariable logistic regression model was performed for each age group to estimate the association between OPA (T1-T2-T3) and positive mental health (T4), controlling for age, gender, puberty stage, and neighborhood income (T1). Linear regression models were also performed to estimate the associations between the exposure (OPA, T1-T2-T3) and mediators (autonomy, competence, relatedness, T3), while logistic regression models were performed to estimate the associations between mediators (T3) and positive mental health (T4), controlling for age, gender, puberty stage, and neighborhood income (T1). These models provide support to conduct mediation analyses. Mediation analyses will only be performed if a statistically significant association is observed between the exposure and the outcome.

**Fig. 1:**
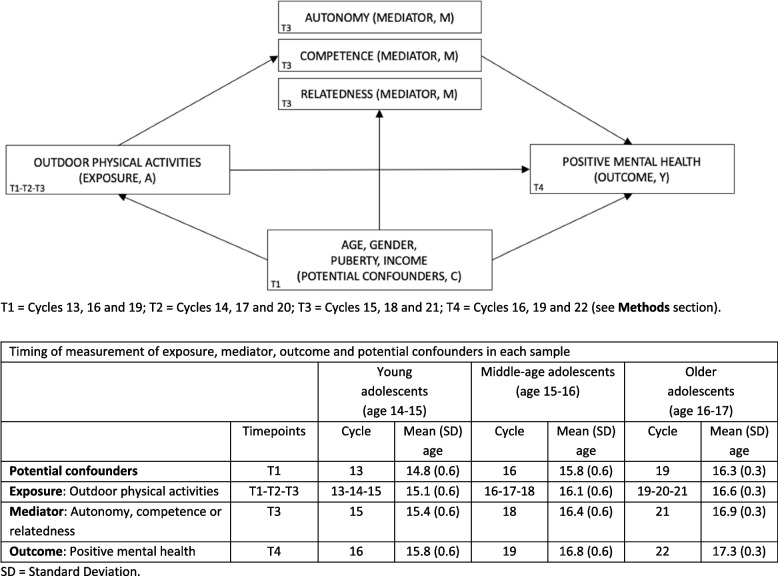
Directed Acyclic Graph representing the potential mediating effect of the satisfaction of the basic psychological needs of autonomy, competence and relatedness on the association between OPA and positive mental health

Mediation analyses within the counterfactual framework allow us to decompose the total effect (TE) between OPA and positive mental health into the natural direct (NDE) and the natural indirect effects (NIE) [67, 70]. NDE is the effect of OPA on positive mental health when the effect through the mediator is blocked. NIE is the effect of the mediator on positive mental health when the exposure is fixed to present (OPA = 1, *high*) to see how much positive mental health would change if satisfaction of autonomy, competence or relatedness increased by 1. In these models, the mediator is set to the potential value it takes in the absence (or reference category) of OPA. Mediation analyses were performed to identify whether autonomy, competence and relatedness (T3), partially or fully mediated the association between OPA (T1-T2-T3) and positive mental health (T4). To minimize the risk of reverse causation bias, we used consecutive (rather than concurrent) measures of confounders (T1), exposure (T1-T2-T3), mediators (T3) and outcome (T4) in all analysis (Fig. 1). In this study, we estimated separate mediation models for each mediator (autonomy, competence, relatedness). Each model tests a distinct hypothetical mechanism specific to the respective mediator. Effects were estimated using bootstrap procedures with percentile confidence intervals, and inference was based primarily on confidence intervals because of their robustness in the presence of asymmetric sampling distributions. For consistency and transparency, we also report standard significance levels (*p* < 0.05, < 0.01, < 0.001). Each model addressed a distinct, theory-driven mechanism and then tested a specific hypothesis; therefore, we did not apply multiple-testing adjustment. According to VanderWeele [68], mediation models should test for interaction between the exposure and mediator, as the presence of exposure–mediator interaction affects the interpretation and decomposition of TE into NDE and NIE. Since none of the interaction terms (e.g., OPA*autonomy) were statistically significant, simplified mediation models that exclude interaction terms are presented. Sensitivity analyses were conducted adding MVPA as a mediator in addition to autonomy, competence or relatedness. More precisely, we calculated sequential mediation analyses with the exposure OPA (T1-T3), MVPA as the first mediator (T3), the respective basic psychological need as the second mediator (T3), and the outcome positive mental health (T4), adjusting for covariates (T1). E-values were computed to examine the robustness to unmeasured confounding [69]. Potential confounders (T1), exposure (T1-T2-T3), mediators (T3) and outcome (T4) were measured at successive data collection to respect temporality and to avoid reverse causation bias. The significance level was set at *p* < 0.05. Statistical analyses were performed using the R Software Version 4.3.0 and mediation analyses were performed using the *CMAverse* package in R.

## Results

For the young adolescent group, a total of 424 participants completed cycle 16. Among them, 392 provided data in at least two of the three data collection cycles over the year (cycles 13–14–15) used to compute the OPA exposure variable and 363 (57.9% female, mean (SD) age at cycle 13 = 14.8 (0.6) years) provided complete data for all study variables and were retained for analyses pertaining to young adolescence. A similar strategy was used to construct the analytical samples for middle-age adolescence (*n* = 270, 58.1% female, mean (SD) age at cycle 16 = 15.8 (0.6) years) and older adolescence (*n* = 129, 59.7% female, mean (SD) age at cycle 19 = 16.3 (0.3) years).

Participants included in the analyses were more numerous among girls in all age groups. Participants included in the analyses were proportionally more likely to have high neighborhood income and less likely to have moderate neighborhood income in all age groups. Participants included and those not included in the analyses had relatively similar puberty stages in young and middle-age adolescence samples, but a relatively higher proportion of those included in the older adolescence sample presented a *postpubertal* stage (Supplementary material Table S1). Based on bivariate descriptive results between OPA frequency continuous measure and positive health scores, we observed an inflection point at the value of 2 OPA frequency, suggesting higher positive mental health scores over this threshold. Therefore, this cutoff was used to distinguish between *low* (0 to 2) and *high (*> 2) OPA frequency. Approximately one third of participants in all age groups reported taking part in high OPA frequency, while approximately half of participants in all age groups reported flourishing mental health (Table 1).

**Table 1:** Characteristics of participants retained for analysis for each group of adolescents

	Young adolescents Cycles 13–16 (*n* = 363)	Middle-age adolescents Cycles 16–19 (*n* = 270)	Older adolescents Cycles 19–22 (*n* = 129)
Variable	n (%),	n (%),	n (%),
	mean (SD) or	mean (SD) or	mean (SD) or
	median (IQR)	median (IQR)	median (IQR)
Gender (female), n (%)	210 (57.9)	157 (58.1)	77 (59.7)
Age (y), mean (SD)	14.8 (0.6)	15.8 (0.6)	16.3 (0.3)
Age (y), range (min–max)	13.8–16.2	14.8–17.8	15.8–16.8
Neighborhood Income tertile grouping, n (%)	-	-	-
High	135 (37.2)	108 (40.0)	60 (46.5)
Moderate	113 (31.1)	75 (27.8)	31 (24.0)
Low	115 (31.7)	87 (32.2)	38 (29.5)
Puberty stage, n (%)	-	-	-
Prepubertal	4 (1.1)	3 (1.1)	0
Early puberty	9 (2.5)	5 (1.9)	5 (3.9)
Midpubertal	56 (15.4)	27 (10.0)	8 (6.2)
Late puberty	236 (65.0)	159 (58.9)	78 (60.5)
Postpubertal	58 (16.0)	76 (28.1)	38 (29.5)
OPA frequency (mean over a year), median (IQR)	1.0 (0.2–3.3)	0.6 (0.1–2.5)	0.3 (0–1.8)
OPA frequency, n (%)	-	-	-
Low OPA	235 (64.7)	189 (70.0)	99 (76.7)
High OPA	128 (35.3)	81 (30.0)	30 (23.3)
Autonomy, mean (SD)	4.8 (1.6)	4.9 (1.6)	5.2 (1.5)
Competence, mean (SD)	5.0 (1.5)	4.9 (1.5)	5.2 (1.5)
Relatedness, mean (SD)	4.7 (1.4)	4.6 (1.4)	5.0 (1.4)
Positive mental health, n (%)	-	-	-
Not flourishing	163 (44.9)	143 (53.0)	61 (47.3)
Flourishing	200 (55.1)	127 (47.0)	68 (52.7)

*SD* Standard Deviation, *IQR* Interquartile Range, *OPA* Outdoor Physical Activity

In multivariable logistic regression analyses, no statistically significant association between OPA frequency and flourishing mental health was found in young adolescents since the 95% confidence intervals (CI) included the null value (OR [95% CI] = 1.10 [0.69, 1.75]). However, a statistically significant positive association was observed in middle-age adolescents (1.99 [1.11, 3.57]) and older adolescents (3.40 [1.25, 10.09]). In linear regression models, high OPA frequency was associated with competence ($\widehat{\beta }$[95% CI] = 0.50 [0.16, 0.84]) and relatedness (0.33 [0.002, 0.65]) in young adolescents and with relatedness (0.57 [0.16, 0.97]) in middle-age adolescents. All estimates of the association between OPA and mediators in older adolescents were none statistically significant since 95% CIs include the null value (Table 2). Finally, all mediators were positively associated with flourishing mental health among all three adolescent age groups (Table 3).

**Table 2:** Beta coefficients, 95% Confidence Intervals (CI) and p-values for the associations between OPA and the satisfaction of the basic psychological needs of autonomy, competence and relatedness among young, middle-age and older adolescents

	**Autonomy**		**Competence**		**Relatedness**	
**OPA frequency**	$\widehat{{\varvec{\beta}}}$**(95% CI)**	***p*-value**	$\widehat{{\varvec{\beta}}}$**(95% CI)**	***p*-value**	$\widehat{{\varvec{\beta}}}$**(95% CI)**	***p*-value**
Young adolescents (*n* = 363)	-		-		-	
Low OPA	ref		ref		ref	
High OPA	0.27 (−0.10, 0.63)	.15	0.50 (0.16, 0.84)**	.004	0.33 (0.002, 0.65)*	.05
Middle-age adolescents (*n* = 270)	-		-		-	
Low OPA	ref		ref		ref	
High OPA	0.35 (−0.09, 0.80)	.12	0.01 (−0.40, 0.42)	.97	0.57 (0.16, 0.97)**	.006
Older adolescents (*n* = 129)	-		-		-	
Low OPA	ref		ref		ref	
High OPA	0.20 (−0.49, 0.89)	.57	0.36 (−0.33, 1.05)	.30	0.03 (−0.61, 0.67)	.93

Models are adjusted for age, gender, puberty stage, and neighborhood income

$\widehat{\beta }$ = Beta coefficient, *CI* Confidence Intervals, *OPA* Outdoor Physical Activity

^*^p <.05

^**^p <.01

^***^p <.001

**Table 3:** Odds ratio (OR), 95% Confidence Intervals (CI) and p-values for the associations between the satisfaction of the basic psychological needs of autonomy, competence and relatedness and positive mental health among young, middle-age and older adolescents

	**Positive mental health**		
	**OR**	**(95% CI)**	***p*-value**
Young adolescents (*n* = 363)	-	-	-
Autonomy	1.66	(1.43, 1.95)***	<.001
Competence	1.73	(1.46, 2.07)***	<.001
Relatedness	1.89	(1.58, 2.29)***	<.001
Middle-age adolescents (*n* = 270)	-	-	-
Autonomy	1.61	(1.34, 1.95)***	<.001
Competence	1.64	(1.35, 2.02)***	<.001
Relatedness	1.95	(1.57, 2.47)***	<.001
Older adolescents (*n* = 129)	-	-	-
Autonomy	1.74	(1.31, 2.40)***	<.001
Competence	1.77	(1.33, 2.43)***	<.001
Relatedness	1.79	(1.30, 2.62)***	<.001

Models are adjusted for age, gender, puberty stage, and neighborhood income

*OR* Odds Ratio, *CI* Confidence Intervals

^*^p <.05

^**^p <.01

^***^p <.001

Mediation analyses were conducted among middle-age and older adolescents only; they were not performed among young adolescents since no statistically significant association was observed between the exposure and the outcome. Also, although causal mediation analysis can take into account the interaction between the exposure and the mediator, interactions were dropped from the analyses, since the interaction terms were not statistically significant. NDE, NIE and TE estimates for all three mediation models are presented in Table 4. Among middle-age adolescents, only the estimate of the NIE for the relatedness mediator excluded the null value (1) suggesting that relatedness mediates the association between OPA and positive mental health. Among older adolescents, all NIE estimates confidence intervals include the null value, which doesn’t support the presence of statistically significant mediated effect through autonomy, competence or relatedness. Results from sensitivity analyses reveal that including MVPA as an additional mediator does not meaningfully change TE, NDE and NIE estimates (Supplementary material Table S2). E-value estimates suggest that our findings are robust to potential unmeasured confounding (Supplementary material Table S3).

**Table 4:** Estimates and 95% Confidence Intervals (CI) of the natural direct, natural indirect and total effects for the association between OPA on positive mental health in single mediator models for autonomy, competence and relatedness, among middle-age adolescents (*n* = 270) and older adolescents (*n* = 129) in the MATCH study

	**Mediator: Autonomy**	**Mediator: Competence**	**Mediator: Relatedness**
	**Estimate (95% CI)**	**Estimate (95% CI)**	**Estimate (95% CI)**
Middle-age adolescents (*n* = 270)	-	-	-
NDE	1.67 (0.99, 3.08)	1.94 (1.16, 3.63)*	1.43 (0.81, 2.98)
NIE	1.15 (0.96, 1.45)	1.00 (0.82, 1.24)	1.37 (1.08, 1.76)**
TE	1.92 (1.11, 3.58)*	1.95 (1.13, 3.63)*	1.95 (1.23, 3.58)*
Older adolescents (*n* = 129)	-	-	-
NDE	2.95 (1.25, 10.75)*	2.65 (1.09, 9.79)*	3.15 (1.23, 11.64)*
NIE	1.10 (0.75, 1.77)	1.19 (0.83, 1.94)	1.02 (0.74, 1.43)
TE	3.26 (1.26, 13.39)*	3.13 (1.23, 13.38)*	3.20 (1.20, 12.46)*

Models include only one mediator (autonomy, competence or relatedness) and are adjusted for age, gender, puberty stage, and neighborhood income. *CI* Confidence Intervals, *NDE* Natural Direct Effect, *NIE* Natural Indirect Effect, *TE* Total Effect

^*^p <.05

^**^p <.01

^***^p <.001

## Discussion

We found a positive association between OPA and positive mental health among middle-age and older adolescents. High OPA is associated with competence and relatedness in young adolescents, and only with relatedness among middle-age adolescents. Autonomy, competence and relatedness are associated with positive mental health in all three groups of adolescents. Finally, we found that relatedness mediates the association between OPA and positive mental health in middle-age adolescents. Taken together, these results contribute to the literature by providing evidence of an association between OPA and positive mental health using a longitudinal design in adolescents. The fact that the association between OPA and positive mental health varies among different age groups of adolescents could reflect that physical activity, nature attributes, or potential benefits may be perceived differently across adolescent stages. In the absence of mediating effects across all mediators (autonomy, competence, relatedness), we hypothesize that OPA might have a different impact on basic psychological needs as mediators compared to other physical activity contexts. This may be explained by different types of nature mechanisms (e.g., attention restoration, stress reduction, biophilia) that could be involved.

This is a novel study investigating associations during adolescence. As such, no studies are directly comparable, although the findings are consistent with those observed in the adult population. The positive association found between OPA and positive mental health in the current study echoes extant literature among adults suggesting that OPA has benefits on mental health [16, 31, 49, 58, 73]. Similarly, studies in adults have found that physical activity in natural environments, compared to indoor physical activity, could offer greater mental health benefits. These benefits can be observed as lower anxiety levels and greater feelings of connection to nature [37], enhanced positive emotions and reduced negative emotions, greater feelings of energy, revitalization and enjoyment [64], as well as positive effects on affect and perceived effort [36].

Our findings suggest that OPA relates to greater satisfaction of psychological needs for competence and relatedness in some adolescent groups. Being physically active outdoors could provide opportunities for creativity, which plays an important role in the cognitive, physical and emotional development of children and can foster competence [22]. However, it remains unclear why this association is observed only for young adolescents in this sample. Also, nature can improve social cohesion and connectedness, as outdoor spaces provide opportunities for interaction with others which would explain benefits of OPA on relatedness [11, 23, 38, 76]. The OPA-relatedness association is also coherent with studies highlighting that the practice of physical activities fosters social interaction and support [28, 45]. This could also refer to the building capacities mechanisms from Markevych’s [42] conceptual model suggesting that exposure to nature, and potentially OPA, could foster social cohesion. However, it remains unclear why no association was found in older adolescents. No association was observed between OPA and autonomy in all three groups of adolescents. However, there is existing evidence suggesting possible mechanisms for fostering mental health by experiencing autonomy i) directly through adventure education programs [59], ii) through increased self-efficacy [52], or iii) through the provision of freedom to explore the surroundings in nature and to choose an activity [9].

Our results indicating that all three basic psychological needs (i.e. autonomy, competence and relatedness) are associated with positive mental health across all age groups are consistent with previous empirical findings [14, 39]. This also aligns with the Self-Determination Theory, suggesting that satisfaction of the basic psychological needs could promote optimal well-being in individuals [55]. Even in the absence of an association between OPA and positive mental health, these findings highlight the potential for interventions supporting mental health by targeting basic psychological needs, regardless of their impact on physical activity, which remains beneficial for adolescents.

In our mediation analyses, only relatedness appears to mediate the association between OPA and positive mental health among middle-age adolescents. Consequently, our hypotheses suggesting that OPA could potentially contribute to more positive mental health through the reinforcement of psychological needs by allowing individuals to decide their own behaviour (autonomy), provide opportunities for accomplishment, achievement, or success (competence) and encourage meaningful connections with others (relatedness) were only partially confirmed. In studies about general physical activity participation, not specific to outdoor contexts, the mediating role of all three basic psychological needs (autonomy, competence, relatedness) has been shown [14, 39]. However, no previous study, to our knowledge, has examined the potential mediating role of satisfaction of the basic psychological needs as mediators of OPA-positive mental health association, which limits comparison and discussion of our results with similar studies. MVPA was not included as a potential confounder in our analyses, as it is located on the causal pathway between OPA and mental health. However, MVPA was included as an additional mediator of the OPA-mental health association in sensitivity analyses, as suggested in previous studies [14] with no meaningful change in the results (Supplementary material Table S2).

### Strengths, limitations and future research

Our use of longitudinal data allowed for multiple measurements over time during adolescence, including successive measurements that respect temporality and avoids reverse causation bias. However, our OPA measure focused on frequency per week and did not consider the duration of the activities. As such, total time spent in OPA should be considered in future research, especially considering that some OPA could be carried out once a week but for a longer duration (e.g., a full day of hiking during the weekend). Consideration of total time spent in OPA could allow comparison of the benefits of practicing an OPA once a week for a longer duration versus several exposures to OPA for shorter time-periods.

Restricted sample size for young adolescents (*n* = 363), middle-age adolescents (*n* = 270) and older adolescents (*n* = 129) may have reduced statistical power to detect effects, which may help explain null results from our mediation analyses. In addition, baseline positive mental health may represent a potential confounder of the associations of interest. However, this variable was not available at T1 for all age groups and was therefore not included in the models. Adjusting for baseline levels would also require assuming that pre-exposure levels of positive mental health are associated with OPA, a relationship for which there is limited theoretical or empirical support. Moreover, selection bias could be present in this study since participants included in the analysis (with complete data) differ from those with missing data. Further, the use of self-report measures involves a risk of information bias, including recall errors and tendencies toward socially desirable responses. Participants represent a single Canadian province (New Brunswick), which may limit generalizability to other youth in Canada.

To advance understanding of the OPA-positive mental health association, future studies should examine self-esteem [46] and physical self-perceptions [41], as they have been identified in the literature as potential mechanisms explaining the mental health benefits of physical activity.

## Conclusion

This study is one of the few studies examining the benefits of OPA on positive mental health at various moments of adolescence and the first assessing the mediating effect of the basic psychological needs of autonomy, competence and relatedness. This contributes to a growing literature on the intersection between OPA and positive mental health in adolescents. Findings from this study offer valuable insights into OPA as a potential strategy to improve positive mental health, particularly in middle-age and older adolescents.

## Supplementary Information

Supplementary Material 1.

## Data Availability

The original data analyzed for this study are available through a data sharing agreement with the MATCH study research team. More information on this may be obtained from the principal investigator of the MATCH study, Dr. Mathieu Bélanger.

## Abbreviations

OPA: Outdoor physical activity
NDE: Natural Direct Effect
NIE: Natural Indirect Effect
TE: Total Effect
MATCH: Monitoring Activities of Teenagers to Comprehend their Habits
MVPA: Moderate-to-vigorous intensity physical activity
